# Hem-1 Complexes Are Essential for Rac Activation, Actin Polymerization, and Myosin Regulation during Neutrophil Chemotaxis

**DOI:** 10.1371/journal.pbio.0040038

**Published:** 2006-01-24

**Authors:** Orion D Weiner, Maike C Rentel, Alex Ott, Glenn E Brown, Mark Jedrychowski, Michael B Yaffe, Steven P Gygi, Lewis C Cantley, Henry R Bourne, Marc W Kirschner

**Affiliations:** **1**Department of Systems Biology, Harvard Medical School, Boston, Massachusetts, United States of America; **2**Department of Cellular and Molecular Pharmacology, University of California, San Francisco, California, United States of America; **3**Center for Cancer Research, Department of Biology, Massachusetts Institute of Technology, Cambridge, Massachusetts, United States of America; **4**Department of Cell Biology and Taplin Biological Mass Spectrometry Facility, Harvard Medical School, Boston, Massachusetts, United States of America; **5**Department of Systems Biology, Harvard Medical School, Division of Signal Transduction, Beth Israel Deaconess Medical Center, Boston, Massachusetts, United States of America; **6**Cardivascular Research Institute, University of California San Francisco, California, United States of America; Adolf-Butenandt-InstitutGermany

## Abstract

Migrating cells need to make different actin assemblies at the cell's leading and trailing edges and to maintain physical separation of signals for these assemblies. This asymmetric control of activities represents one important form of cell polarity. There are significant gaps in our understanding of the components involved in generating and maintaining polarity during chemotaxis. Here we characterize a family of complexes (which we term leading edge complexes), scaffolded by hematopoietic protein 1 (Hem-1), that organize the neutrophil's leading edge. The Wiskott-Aldrich syndrome protein family Verprolin-homologous protein (WAVE)2 complex, which mediates activation of actin polymerization by Rac, is only one member of this family. A subset of these leading edge complexes are biochemically separable from the WAVE2 complex and contain a diverse set of potential polarity-regulating proteins. RNA interference–mediated knockdown of Hem-1–containing complexes in neutrophil-like cells: (a) dramatically impairs attractant-induced actin polymerization, polarity, and chemotaxis; (b) substantially weakens Rac activation and phosphatidylinositol-(3,4,5)-*tris*-phosphate production, disrupting the (phosphatidylinositol-(3,4,5)-*tris*-phosphate)/Rac/F-actin–mediated feedback circuit that organizes the leading edge; and (c) prevents exclusion of activated myosin from the leading edge, perhaps by misregulating leading edge complexes that contain inhibitors of the Rho-actomyosin pathway. Taken together, these observations show that versatile Hem-1–containing complexes coordinate diverse regulatory signals at the leading edge of polarized neutrophils, including but not confined to those involving WAVE2-dependent actin polymerization.

## Abstract 2

The authors describe a group of multiprotein complexes, scaffolded by Hem-1 (a subset of which also contain WAVE2), that are involved in generating and maintaining cellular polarity in neutrophils.

## Introduction

Directed cell polarity is necessary for unicellular organisms to hunt and mate, for metazoan development, and for leukocytes to mediate inflammation and immune responses. Migrating cells need to make different actin assemblies at the front and the back and to maintain physical separation of signals for these assemblies. This exquisitely coordinated control of asymmetric morphology and regulatory signals represents one important form of cell polarity. Cells can polarize directionally in response to very subtle spatial cues (e.g., gradients of extracellular chemoattractants), and some polarize even in response to uniform stimulation [1].

The linear cascades known to connect chemoattractant receptors to the cytoskeleton do not suffice to explain polarity. Instead, this self-organizing process presumably requires modulation of these primary circuits by positive and negative feedback loops, mediated by specifically localized complexes of regulatory proteins. Using biochemistry and reverse genetics, we have identified a family of such complexes that share a common regulatory scaffold and appear to coordinate these circuits at the leading edge of neutrophils.

The highly conserved polarity circuits of yeast, *Dictyostelium,* and neutrophils [2–5] all use positive feedback loops, controlled by a Rho-family GTPase, to stabilize the leading edge. In neutrophils, Rac plays key roles in two positive feedback circuits (Rac/phosphatidylinositol-(3,4,5)-*tris*-phosphate [PIP_3_]/actin positive feedback loop) that organize the leading edge and drive stable protrusion [6–11]. A related GTPase, Rho, organizes myosin-based contraction at the trailing edge, primarily by inactivating myosin light chain phosphatase and inducing local phosphorylation and activation of myosin light chains [12]. Local inhibitions by Rac and Rho of one another's activities are essential for proper organization of polarity [13].

Rac has consistently emerged as one of the most important proteins regulating polarity and cytoskeletal rearrangements during chemotaxis [14–18]. Proteins of the WAVE/suppressor of cyclic AMP receptor (SCAR) family mediate Rac-induced actin polymerization and are necessary for actin rearrangements and cell motility in many different contexts [19–31]. Rac does not directly associate with WAVE but interacts with WAVE via a stable multimeric complex of proteins that includes 121F-specific p53 inducible RNA (PIR121)/Sra-1/cytoplasmic fragile X mental retardation interacting protein (CYFIP), hematopoietic protein (Hem)-2/Nck-associated protein 125 kDa (Nap125), E3b1/Abelson interactor (Abi), and Hspc300 [32] (see Table 1 for nomenclature of WAVE complex subunits in various species). In contrast to a related family member, Wiskott-Aldrich syndrome protein (WASP), which is *auto*-inhibited through intramolecular interactions [33], WAVE is *trans*-inhibited through association with other proteins in the WAVE complex [32]. The heterodimer of CYFIP1/PIR121 and Hem-2/Nap125 can form a stable subcomplex [34] and links the rest of the complex to Rac [35] and potentially to Nck [36,37]. We refer to the Hem-1/Hem-2/Nap125 + CYFIP1/2 heterodimer as the WAVE regulatory complex (Table 1).

**Table 1:**
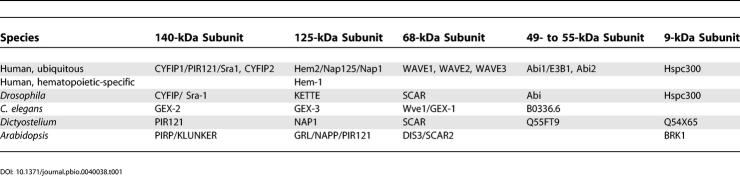
WAVE Complex Homologs and Nomenclature in Various Species

Based on the *trans*-inhibitory mode of WAVE regulation, we hypothesized that the WAVE regulatory complex scaffold might also control effectors other than WAVE2 downstream of Rac. We focused on leukocyte cells and lysates for these experiments, as hematopoietic cells have represented a highly abundant source for other effectors of chemoattractant signaling [38–41]. To look for cognate complexes lacking WAVE but potentially capable of regulating other effectors downstream of Rac, we focused on a putative subunit of the leukocyte WAVE complex, Hem-1, which was initially identified as a transcript specifically expressed in hematopoietic cells [42]. Based on its homology to a conserved protein component (Hem-2/Nap125) found in WAVE regulatory complexes, we postulated that Hem-1 would be present in the leukocyte WAVE regulatory complex. Homologs of Hem-1 (Hem-2/Nap125) are necessary for cell migration and actin polymerization in Caenorhabditis elegans, *Arabidopsis*, *Drosophila*, and mouse melanoma cells [23,24,43–49].

We find that Hem-1–scaffolded complexes do not partition exclusively with WAVE but instead include numerous other proteins that regulate polarity. Hem-1 localizes to the leading edge of polarized neutrophils, and Hem-1 complexes are required for polarized actin polymerization, for positive feedback loops that stabilize the leading edge, and for excluding myosin phosphorylation from the leading edge.

## Results

### Hem-1 Localizes to the Leading Edge of Polarized Neutrophils

**Figure 1:**
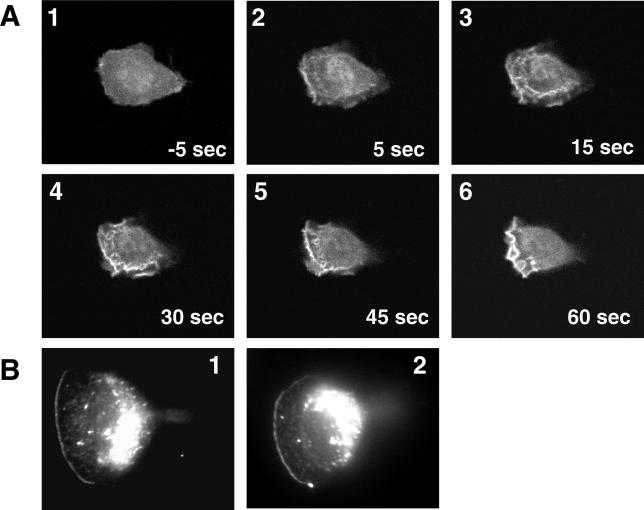
Hem-1 Strongly Polarizes following Uniform Stimulation of HL-60 cells (A) Differentiated HL-60 cells expressing GFP-tagged Hem-1 were exposed to uniform 20 nM fMLP (at t = 0 s) and imaged with a spinning disk confocal microscope at 5-s intervals at 37 °C. See Video S1. (B) Human neutrophils obtained from finger pinprick [80] were exposed to uniform 20 nM fMLP for 3 min, fixed, and immunostained for endogenous Hem-1. Hem-1 immunostaining is observed at the leading edge of polarized cells (and also bright staining in the cytosol).

Hem-1′s subcellular location is ideal for scaffolded complexes that control leading edge activities during chemotaxis, as shown in experiments with green fluorescent protein (GFP)-tagged Hem-1 expressed in differentiated HL-60 (dHL-60) cells, a neutrophil-like cell line. GFP-Hem-1, found throughout the cytosol of unstimulated cells, rapidly translocates to the cell periphery on exposure to a uniform concentration of a tripeptide chemoattractant, formyl-methionyl-leucyl-phenylalanine (fMLP). As the cells polarize, Hem-1 goes on to accumulate almost exclusively at the leading edge, with spatial and temporal dynamics similar to those of ruffling and protrusion of the pseudopod (Figure 1A and Video S1). Endogenous Hem-1 also concentrates at the leading edge of polarized neutrophils (Figure 1B) as well as intracellular puncta. These puncta are not observed for GFP-tagged Hem-1 and may represent the cytosolic pool of Hem-1 complexes.

### The Hem-1 Subunit of the WAVE Regulatory Complex Exists in Pools outside of the WAVE2 Complex

The *trans*-inhibitory mode of WAVE regulation raised the possibility that Hem-1–containing complexes might also control effectors other than WAVE2. If so, Hem-1 complexes should exist in biochemical pools distinct from WAVE2, be more abundant than WAVE2, and associate with proteins other than or in addition to WAVE2. All these predictions were confirmed in leukocyte lysates.

**Figure 2:**
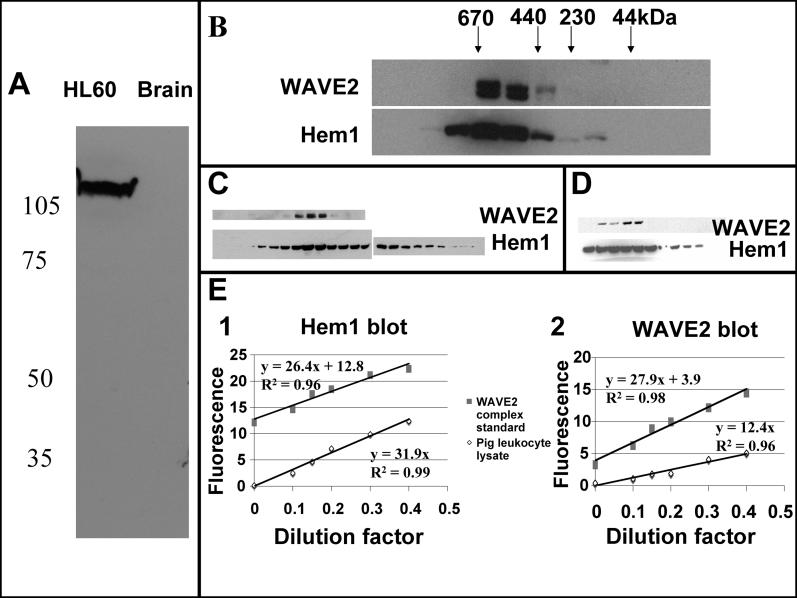
Hem-1 Component of WAVE Regulatory Complex Exists in Pools outside the WAVE2 Complex (A) Hem-1 antibody specifically recognizes Hem-1 and not the more ubiquitous Hem2/Nap125 homolog. Western blot of equal amounts of differentiated HL-60 (human neutrophil-like cell) and mouse brain lysate with antibodies directed against Hem-1. The Hem-1 antibody also fails to react against 293 lysates (data not shown). (B) Gel filtration of neutrophil-like HL-60 cell lysate blotted for Hem-1 and WAVE2. Note that both proteins migrate primarily as large complexes that generally cofractionate, but Hem-1 is also present in high-molecular-weight fractions that are devoid of WAVE2. The positions of molecular weight standards (thyroglobin, ferritin, catalase, and albumin) are noted. (C, D) Chromatograms of leukocyte lysate during WAVE2 complex purification. The WAVE2 complex was purified from pig leukocytes using conventional chromatography and antibodies to WAVE2 to determine which fractions to pool. (C) HiTrap S chromatogram (of 0% to 40% ammonium sulfate cut of pig leukocyte lysate) blotted for Hem-1 and WAVE2. Note a broader distribution of Hem-1 versus WAVE2. (D) HiTrap Q chromatogram (of the peak WAVE2 fractions from the HiTrap S column) blotted for Hem-1 and WAVE2. Note a broader distribution of Hem-1 versus WAVE2. Subsequent chromatography of the WAVE2-reactive fractions from the Q column yields superimposable profiles for Hem-1 and WAVE2 are (data not shown). (E) Quantitation of Hem-1 and WAVE2 in pig leukocyte lysates. Partially purified WAVE2 complex was used to quantitate relative concentrations of Hem-1 and WAVE2 in pig leukocyte lysates. The ratio of all of the components of the WAVE2 complex is 1:1 in the purified complex. Protein concentrations can be determined by fitting a single point to a standard curve or by comparing two dilution series to one another. The latter is a more accurate means to calculate protein concentration. When plotted from 0× to 1× dilution on the x-axis, the ratio of the slopes of two dilution series indicates their relative concentrations. The WAVE2 complex was serially diluted into pig leukocyte lysate (top curves—dilution into lysate is necessary because some proteins blot differently in buffer versus lysate) or pig leukocyte lysate was serially diluted into buffer (bottom curves). Both blots (Hem-1 and WAVE2) are from the same dilution series and the same gel. For this experiment, 14.8 nM WAVE2 complex standard and 1:20 pig leukocyte lysate represent 1× dilution (from which the 0.1×, 0.2×, 0.3×, and 0.4× dilutions were prepared). The relative slopes of the curves in each blot indicate concentration of proteins in the lysate versus the WAVE2 standard. Thus, for this experiment, the Hem-1 concentration in pig leukocyte lysate (E1) is 31.9/26.4 or 1.2× the concentration of the WAVE2 complex standard. Thus, 1.2 × 14.8 (concentration of Hem1 in Wave2 complex standard) × 20 (dilution factor of pig lysate) = approximately 360 nM Hem-1 in a 1× pig leukocyte lysate. Similarly, WAVE2 (E2) is 12.4/27.9 or 0.45× the concentration of the WAVE2 complex standard. Thus, 0.45 × 14.8 (concentration of WAVE2 in 2 complex standard) × 20 (dilution factor of pig lysate) = approximately 130 nM WAVE2 in a 1× pig leukocyte lysate. Curves represent averages of experiments performed in duplicate.

Pools of Hem-1 can be biochemically separated from the WAVE2 complex. Immunoblotting with antibodies raised against Hem-1 (Figure 2A) showed that essentially all Hem-1 in neutrophil lysates fractionates by gel filtration in large complexes centered at about 600 kDa (Hem-1 is only 128 kDa) (Figure 2B). Hem-1 and WAVE2 partially overlap in eluates from gel filtration columns, but the Hem-1 peak is broader than that of WAVE2, and high-molecular-weight pools of Hem-1 are found in fractions devoid of WAVE2. Hem-1 similarly shows a much broader biochemical profile than WAVE2 when neutrophil lysates are subjected to chromatography on two different ion exchange columns (Figure 2C and 2D).

**Figure 3:**
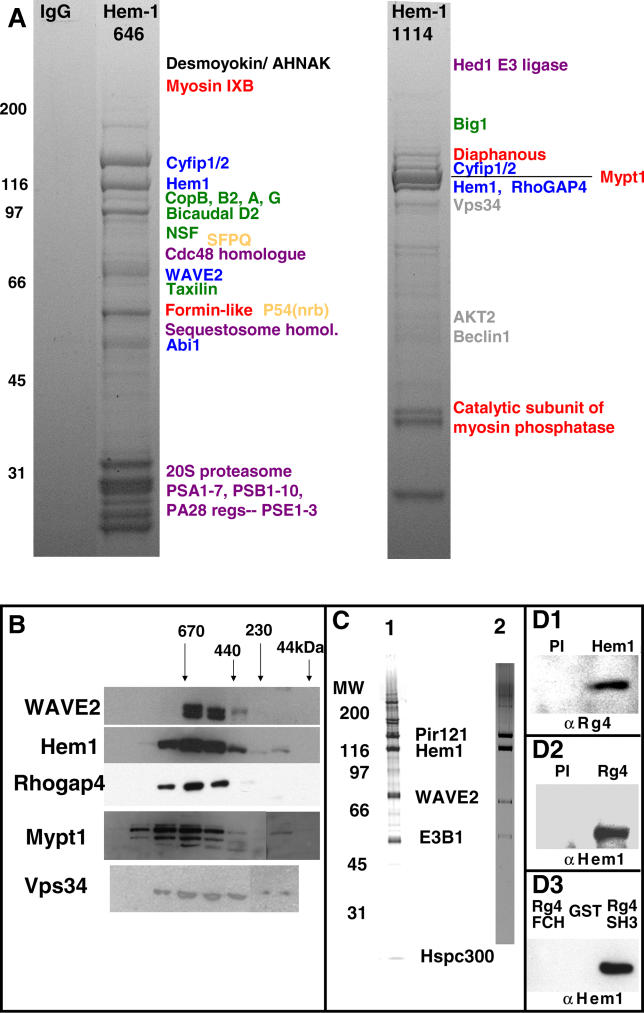
Antibodies to the Hem-1 Component of the WAVE Regulatory Complex Immunoprecipitate Potential Polarity Effectors in Addition to the WAVE2 Complex (A) Two milliliters of neutrophil-like differentiated HL-60 lysate (containing 10 mg of protein) were immunoprecipitated with one of two Hem-1 antibodies, raised against an internal region of Hem-1 (646–659) or the C-terminus of Hem-1 (1114–1127), and eluted with the corresponding peptides. The same immunoprecipitation and elution were performed with purified rabbit IgG as a control. The gel was stained with Gelcode Blue. Mass spectrometry–based protein IDs are noted at approximate gel slice position. Colors of proteins indicate major functional classes. Blue, expected components of WAVE2 complex; red, regulators of actin and myosin; green, proteins implicated in vesicle or mRNA trafficking; gray, proteins implicated in lipid signaling; yellow, splicing factors; and purple, proteins implicated in protein degradation. (B) Several of the proteins that coimmunoprecipitate with Hem-1 run as large protein complexes. Crude HL-60 lysate was analyzed by gel filtration on Superose 6 and blotted with antibodies to Hem-1, WAVE2, RhoGAP4, the regulatory subunit of myosin light chain phosphatase (Mypt1), or Vps34 (class III PI3K). The positions of molecular weight standards (thyroglobin, ferritin, catalase, and albumin) are noted. (C) Silver-stained SDS-PAGE of Superose 6 peak of conventionally purified native pig leukocyte WAVE2 complex, including protein identifications (C1). All proteins were identified via mass spectrometry with the exception of Hspc300. Alternatively, antibodies to Hem-1 (646–659) were used to immunoprecipitate the WAVE2 complex from the Superose 6 WAVE2 peak to unambiguously distinguish WAVE2 components from remaining contaminants (C2). Besides the expected core components of the WAVE2 complex, no other Hem-1–associated proteins were identified via mass spectrometry in the purified native pig leukocyte WAVE2 complex. (D) Endogenous Hem-1 and RhoGAP4 reciprocally immunoprecipitate. (1) HL-60 lysates were immunoprecipitated with either Hem-1 or preimmune antisera, eluted, and blotted with RhoGAP4 antibodies. RhoGAP4 signal represents approximately 1% of input. (2) HL-60 lysates were immunoprecipitated with either RhoGAP4 or preimmune antisera, eluted, and blotted with Hem-1 antibodies. Hem-1 signal represents approximately 1% of input. (3) The SH3 domain of RhoGAP4 is sufficient for interaction with Hem-1. GST-tagged RhoGAP4 SH3 was incubated with HL-60 lysate, eluted with glutathione, and blotted for Hem-1. The FCH domain of RhoGAP4 and GST alone fail to pull down Hem-1 from lysates.

If Hem-1 is present in complexes in addition to the WAVE2 complex, its cellular abundance should be greater than that of WAVE2. We quantitated the relative concentrations of Hem-1 and WAVE2 in leukocyte cytosol, using native purified pig leukocyte WAVE2 complex as a standard; the standard contains equimolar concentrations of Hem-1 and WAVE2. Serial dilutions of the WAVE2 complex versus leukocyte cytosol reveal a 2.8-fold greater abundance of Hem-1 than of WAVE2 (360 versus 130 nM) in undiluted leukocyte lysate (Figure 2E), indicating that more than 60% of Hem-1 is not associated with WAVE2. Excess Hem-1 is associated with other proteins, because the Hem-1 in crude lysates migrates on gel filtration in complexes at least as large as the WAVE2 complex (Figure 2B). Moreover, pools of Hem-1 in excess of WAVE2 are unlikely to associate primarily with other WAVE isoforms, because only WAVE2 is identified in Hem-1 immunoprecipitates from HL-60 cells (Figure 3A) and only trace amounts of WAVE1 and WAVE3 protein are present in HL60 cells (Figure S1). Taken together, these data strongly suggest that Hem-1 forms large complexes with proteins other than WAVE2.

### Putative Polarity Regulators Coimmunoprecipitate with Hem-1

To identify possible non-WAVE effectors for the regulatory subunits of the WAVE complex, we used peptide antibodies to immunoprecipitate endogenous Hem-1 from neutrophil-like HL-60 lysate (Figure 3A). Antibodies raised against an internal or a C-terminal Hem-1 peptide both effectively precipitated Hem-1 along with CYFIP1/PIR121 and its homolog CYFIP2 (Figure 3A), which are usually associated with Hem-1 homologs (see Table 1). Only the antibody directed against the internal Hem-1 epitope coimmunoprecipitated the other known components of the WAVE2 complex (indicated by blue in Figure 3A), suggesting that different Hem-1 epitopes are exposed in different protein complexes. In contrast, no detectable proteins are immunoprecipitated with control IgG under these conditions.

In addition to the expected components of the neutrophil WAVE2 complex, both Hem-1 antibodies also immunoprecipitate several excellent candidates for regulating the positive and negative feedback loops involved in leukocyte polarity. In particular, Hem-1 coimmunoprecipitates with proteins that could play a role in excluding myosin activity from the leading edge, including myosin light chain phosphatase (the catalytic subunit and the myosin phosphatase targeting subunit Mypt1) and two different Rho-specific GTPase activating proteins (GAPs)—RhoGAP4 and myosin IXB (which is a myosin motor and a Rho-specific GAP [50]) (see Discussion). Furthermore, Hem-1 associates with proteins that could play a role in front positive feedback, including Vps34 and Abi-1 (see Discussion). The proteins unambiguously identified by mass spectrometry in Hem-1 immunoprecipitations are shown at their relative positions in (Figure 3A).

The most abundant coimmunoprecipitating proteins could be identified in small-scale immunoprecipitations from 300 μl of neutrophil cytosol; these include RhoGAP4 (a hematopoietic Rho-specific GAP [51,52]), myosin light chain phosphatase, and Vps34, a class III phosphatidylinositol-3′-kinase (PI3K). Like WAVE2, these proteins comigrate with Hem-1 as high-molecular-weight complexes on gel filtration (Figure 3B), suggesting that a substantial portion of these proteins could be associated with some of the same regulatory subunits (e.g., Hem-1 and CYFIP1/2) that are also found in the WAVE complex. Indeed, knockdown of Hem-1 decreases the amount of RhoGAP4 present in high-molecular-weight gel filtration fractions, suggesting a significant partitioning of RhoGAP4 in Hem-1 complexes (Figure S2). These novel leading edge complexes are unlikely, however, to contain WAVE2, which can be separated from them with conventional chromatography (Figure 3C).

Additional evidence suggests that RhoGAP4, myosin light chain phosphatase, and Vps34 are associated with Hem-1–scaffolded complexes distinct from the WAVE2 complex. For example, myosin light chain phosphatase, WAVE2, and RhoGAP4 exhibit different elution profiles from one another on cation and anion exchange columns (data not shown). Moreover, Hem-1 antibodies immunoprecipitated RhoGAP4 from lysates of dHL-60 cells (Figure 3D1), and antibodies generated against RhoGAP4 immunoprecipitated Hem-1 (Figure 3D2), but not WAVE2 (data not shown). Although we cannot exclude the possibility that Rhogap4, WAVE2, and other components are present in larger complexes that do not survive immunoprecipitation and ion exchange, we favor the interpretation that they represent separate Hem-1 complexes. Three proteins that associate with Hem-1/Hem-2—including Abi-1 (one of the subunits of the WAVE2 complex), Nck (an SH2/SH3 domain containing adaptor protein that is known to bind the Hem-1 homolog Hem-2/Nap125), and RhoGAP4—contain highly homologous SH3 domains, which may represent a general binding motif for components of the leading edge complexes. Indeed, the SH3 domain of RhoGAP4 sufficed to pull down Hem-1 from neutrophil lysates (Figure 3D3).

### Actin Polymerization and Polarity Require Hem-1

**Figure 4:**
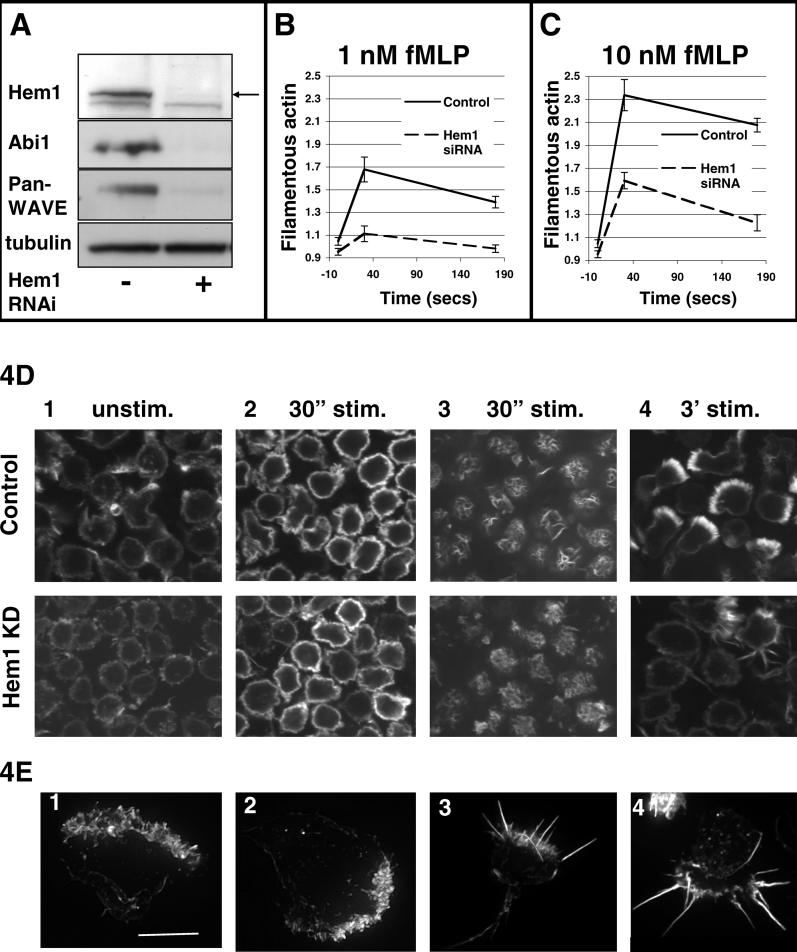
Actin Polymerization and Polarity Are Dependent on Hem-1 (A) RNAi-mediated inhibition of endogenous Hem-1 protein expression and degradation of WAVE complex proteins in HL-60 cells. Whole cell lysates were prepared from control cells and siRNA-expressing cells as described. Equal amounts of lysate were used to determine expression of Hem-1, Abi-1, and pan-WAVE by Western blotting. Tubulin levels are shown as a loading control. (B, C) Actin polymerization is dependent on Hem-1. Differentiated HL-60 cells were treated with or without 1 nM fMLP (B) or 10 nM fMLP (C) for 30 s or 3 min, as indicated. After fixation, F-actin content was assessed by staining with Alexa647-conjugated phalloidin and quantified by FACS analysis. Absolute actin levels are shown for each condition (averages of control and Hem-1 knockdown cell Alexa 647 staining was used to normalize for FACS variation and staining intensity between experiments). Standard error of mean for six experiments is shown. (D) Cell polarity is dependent on Hem-1. Control and Hem-1 knockdown cells were stimulated in suspension with or without uniform fMLP (20 nM final concentration) for 30 s or 3 min, fixed and stained with rhodamine phalloidin to visualize filamentous actin, and visualized with spinning disk confocal microscopy. Unstimulated cells are nonpolar with minimal filamentous actin accumulation (1). Both control and knockdown cells uniformly accumulate F-actin at 30 s following stimulation (2). However, knockdown cells exhibit a more diffuse actin accumulation than control cells (3 is focal plane at top surface of cells in 2). Following 3 min of stimulation (4), 80% of wild-type cells polarize (1,106 of 1,387), compared with only 19% of Hem-1 knockdown cells (291 of 1,544). (E) siRNA-mediated Hem-1 silencing alters the morphology of the leading edge. Adherent cells were stimulated by a uniform concentration of fMLP (10 nM) for 3 min, fixed, and stained with rhodamine-conjugated phalloidin. Fluorescent images were captured as described. (1, 2) Control cells. (3, 4) Hem-1 knockdown cells. Fifty-five percent of Hem-1 knockdown cells (110 of 200) and 4.4% of control cells (11 of 248) exhibit spikes at the leading edge.

To assess potential roles of leading edge complexes in cell polarity, we used RNA interference (RNAi) to deplete Hem-1 in dHL-60 cells (Figure 4A). As previously shown for the genetic ablation or RNAi of the WAVE regulatory complex in other systems [22–24,49,53], depletion of Hem-1 leads to substantial decreases in cellular content of other WAVE2 complex components, including Abi-1 and WAVE (Figure 4A).

Figure 4 also shows that Hem-1 is required for actin polymerization, cell polarity, and morphologic organization of the leading edge. As expected for cells depleted of WAVE2, loss of Hem-1 produced dramatic defects in actin polymerization (Figure 4B and 4C); these defects were more severe at 3 min than at 30 s and at a subsaturating concentration of fMLP (1 versus 10 nM; Figure 4B versus 4C). Moreover, the cellular distribution of polymerized filamentous actin (F-actin) differs markedly in control versus Hem-1–depleted cells treated in suspension with fMLP (Figure 4D). Unstimulated cells have relatively low levels of cortical F-actin (Figure 4D1). At a high concentration of fMLP (20 nM), both control and Hem-1–depleted cells accumulate F-actin 30 s following stimulation (Figure 4D2), but control cells organize F-actin into discrete ruffles, while F-actin is more diffusely distributed in Hem-1–depleted cells (Figure 4D3). At 3 min following stimulation, 80% of control cells (1,106 of 1,387) are clearly polarized, with strong F-actin accumulation at the leading edge (Figure 4D4). In contrast, only 19% of Hem-1–depleted cells (291 of 1,544) polarize under the same conditions, and those that do polarize exhibit aberrant morphology, with long thin actin spikes protruding at the leading edge (Figure 4E).

### Rac Activation and PIP_3_ Production Are Dependent on Hem-1

**Figure 5:**
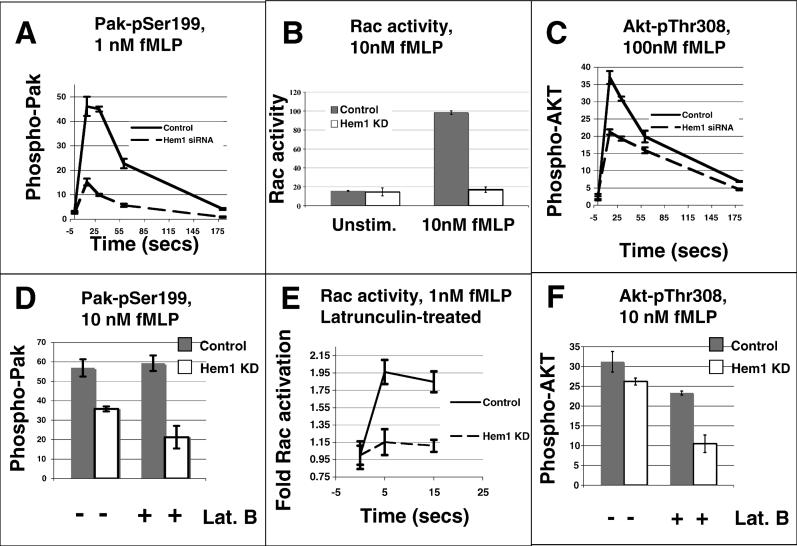
Rac Activation and PIP_3_ Generation Are Dependent on Hem-1 Control or Hem-1 knockdown cells were stimulated in suspension with the indicated dose of agonist. TCA was used to stop the reaction (A, C, D, F) and precipitate proteins for immunoblotting or cells were lysed with NP-40, and Rac-GTP was captured with the Rac-binding domain of PAK (B, E). Western blots were quantitated using fluorescent secondary antibodies and the Odyssey system. The y-axis indicates integrated (background-subtracted) fluorescence of secondary antibody on blots in arbitrary units. All experiments were performed in triplicate and are shown with standard error of mean. (A) Effect of Hem-1 depletion on phosphorylation of PAK (readout of Rac activation) for cells treated with 1 nM fMLP. (B) Effect of Hem-1 depletion on Rac activation (assayed with PAK GBD pulldown) for cells treated with 10 nM fMLP for 15 s. (C) Effect of Hem-1 depletion on phosphorylation of Akt/PKB (readout of PIP_3_ production) for cells stimulated with 100 nM fMLP. (D–F) Defects in PIP_3_ generation and Rac activation are independent of actin polymerization. Results are shown with standard error for cells stimulated in triplicate at 37 °C. (D) Cells were preincubated with 10 μM latrunculin B, stimulated with 10 nM fMLP for 30 s, and blotted with antibodies to PAK-phospho-Ser199. (E) Cells were preincubated with 10 μM latrunculin, stimulated with 1 nM fMLP for 15 s, lysed, and incubated with PAK-GBD (to capture Rac-GTP), and blotted for Rac. (F) Cells were preincubated with 10 μM latrunculin B, stimulated with 10 nM fMLP for 15 s, and then blotted with antibodies to Akt/PKB phospho-Thr308.

The proteins that associate with Hem-1 led us to suspect that leading edge complexes play roles in the Rac/actin/PIP_3_ positive feedback loop [6,8–11] that organizes the leading edge. We used two assays to measure Rac activity. We measured phosphorylation of p21-activated kinase (PAK), which depends on Rac1 in macrophages [54] and serves as a readout for active Rac in leukocytes [39]. Alternatively, we used a PAK GTPase binding domain (GBD) pulldown assay, which measured Rac-GTP levels in cell lysates [55]. We stimulated cells in suspension with chemoattractant, fixed them with trichloroacetic acid (TCA), and analyzed total cell lysates with phospho-specific antibodies or cells were lysed with NP40, and Rac-GTP was captured with the Rac-binding domain of PAK (Figure 5). PAK phosphorylation is significantly diminished in Hem-1–depleted cells (Figure 5A) especially at low fMLP concentrations (78% decrease 30 s after stimulation with 1 nM chemoattractant). Using a PAK GBD pulldown assay, Hem-1–depleted cells failed to increase Rac-GTP in response to chemoattractant (Figure 5B). As an indirect assay of PIP_3_ generation, we measured phosphorylation of Akt/protein kinase B (PKB), a kinase that is activated by PIP_3_ [56–58]. Akt/PKB phosphorylation is decreased by 43% at 15 s after exposure of Hem-1–depleted cells to fMLP (Figure 5C), indicating that Hem-1 is necessary for full chemoattractant-induced PIP_3_ production.

These observations suggest that leading edge complexes participate in the Rac/actin/ PIP_3_ positive feedback loop that organizes the leading edge. The importance of F-actin in this loop [9] and the profound defects in F-actin distribution induced by Hem-1 depletion (Figure 4) raise the possibility that decreased Rac activation and Akt/PKB phosphorylation simply reflect the requirement of Hem-1–containing complexes for actin polymerization. To test the idea that Hem-1–containing leading edge complexes support the feedback loop by mechanisms independent of WAVE2-dependent actin polymerization, we treated control or Hem-1–depleted cells with latrunculin B, which sequesters monomeric actin [59,60] and blocks chemoattractant-induced actin polymerization in neutrophils [9,61,62]. Hem-1 depletion proved to inhibit fMLP-stimulated PAK phosphorylation (Figure 5D), Rac activation (Figure 5E), and Akt/PKB phosphorylation (Figure 5F) in latrunculin-treated cells to an even greater degree than in untreated cells. We infer that leading edge complexes promote fMLP-stimulated Rac activation and PIP_3_ production via mechanisms independent of and in addition to the WAVE complex's role in controlling actin assembly.

### Not All Chemoattractant Pathways Require Hem-1

**Figure 6:**
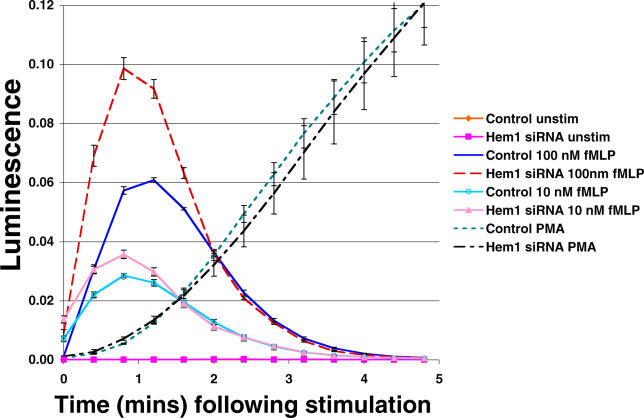
Hem-1 Knockdown Does Not Inhibit Reactive Oxygen Production in Response to Chemoattractant The 6- to 7-day differentiated HL-60 cells were stimulated with the indicated concentration of fMLP or PMA, and reactive oxygen production was monitored in a luminometer. (Control unstimulated cells are superimposable with Hem-1 siRNA unstimulated cells.) Results are shown with standard error for cells stimulated in triplicate at 37 °C.

RNAi directed against Hem-1 does not nonspecifically inhibit all chemotactic signals (Figure 6). We assessed the effects of fMLP on superoxide production, a response that is mediated by Rac and PIP_3_ [63–65] but is independent of polarity and migration. Phorbol ester, which activates superoxide independent of chemoattractant receptors, is indistinguishable in control and Hem-1–depleted cells, indicating that the RNAi did not nonspecifically impair the cells' superoxide-producing machinery. Stimulation of superoxide production by fMLP (10 nM) was not significantly impaired by Hem-1 depletion, and a higher fMLP concentration (100 nM) induced a 67% greater peak superoxide response in the Hem-1–depleted cells (Figure 6). The enhanced superoxide response may reflect the actin polymerization defect of Hem-1–depleted cells, because actin depolymerization reportedly potentiates superoxide production [66]. Indeed, latrunculin-induced depolymerization of F-actin abrogates the difference in reactive oxygen production between control and Hem-1 knockdown cells (Figure S3).

### Chemotaxis, Motility, and Myosin Regulation Depend on Hem-1

**Figure 7:**
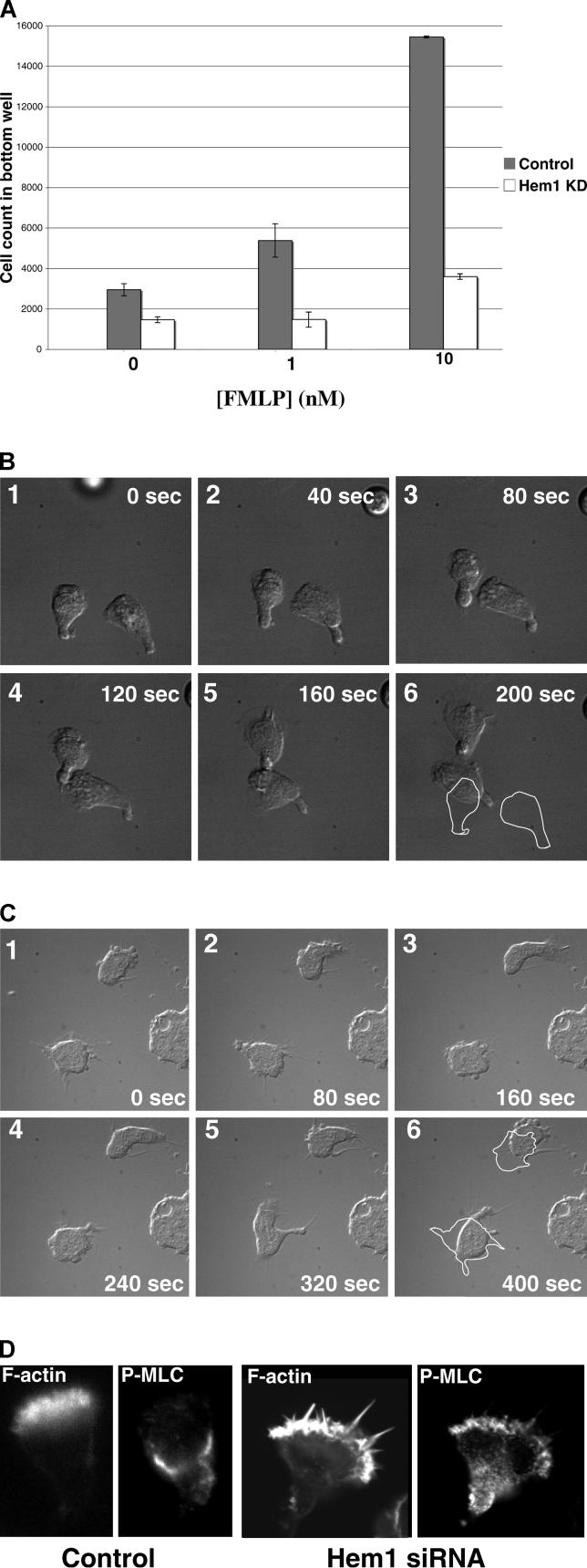
Chemotaxis, Stable Polarity, and the Spatial Regulation of Myosin Phosphorylation Are Dependent on Hem-1 (A) Equal numbers of differentiated control or Hem-1 knockdown cells were placed in the top of a Boyden chamber with the indicated concentration of chemoattractant in the bottom chamber. The y-axis indicates count of cells in bottom (chemoattractant) well at 3 h following stimulation. Results are shown with standard error for experiment performed in triplicate. (B,C) Time-lapse Nomarski images of control (B, Video S2) or Hem-1 knockdown (C, Video S3, Video S4) cells sandwiched between two coverslips and exposed to uniform 20 nM fMLP. Last frame shows outline of cells from first frame. Time in seconds from first frame (cells were prestimulated for 3 to 5 min prior to imaging). (D) Hem-1-knockdown cells fail to properly inhibit myosin light chain phosphorylation at the leading edge. Control or Hem-1 knockdown cells were stimulated with 20 nM fMLP for 3 min, fixed, and stained with fluorescent phalloidin to visualize filamentous actin or with antibodies to phosphorylated myosin light chain (phospho-Ser19). Representative staining for polarized cells is shown.

As expected from the essential role of Hem-1–containing leading edge complexes in actin assembly, cell polarity, and chemotactic signals (Rac activation and PIP_3_ production), depletion of Hem-1 dramatically impaired chemotaxis. Transwell assays, which measure the ability of cells to migrate from buffer through a filter to buffer containing chemoattractant, showed significantly defective chemotaxis in Hem-1–depleted cells at 10 nM fMLP and no stimulated migration at all at a lower concentration, 1 nM (Figure 7A).

Hem-1 depletion strikingly disrupt fMLP-stimulated polarity and migration assessed in a “chimney assay,” in which a small volume of cells is sandwiched between two coverslips [67]. We used this assay, rather than examining cells that adhere to a fibronectin-coated coverslip before addition of fMLP, because Hem-1–depleted cells exhibit a marked adhesion defect (not shown); the chimney assay, which does not require cells to adhere to the coverslips, shows migratory behavior of an unbiased population of adherent and nonadherent cells rather than the small number of Hem-1 knockdown cells that do adhere to fibronectin. Uniform chemoattractant induces control cells in the chimney assay to polarize and migrate rapidly and persistently; polarity is maintained for many minutes (Figure 7B and Video S2). In contrast, most Hem-1 knockdown cells (Figure 7C and Videos S3 and S4) fail to polarize, often blebbing or forming spikes uniformly along their periphery. Cells that do polarize do so only transiently, and then they rapidly retract their leading edges back into the cell body; their labile fronts and backs constantly change places, in contrast to the persistent fronts and backs of control cells.

Several Hem-1–associated proteins could play a role in inhibiting myosin-based contraction at the leading edge (Figure 3A). If so, Hem-1 knockdown cells would be expected to accumulate hyperactivated (hyperphosphorylated) myosin light chain at the leading edge. Figure 7D confirms this prediction: Hem-1 depletion causes phosphorylated myosin light chain to accumulate at both the leading and trailing edges, in contrast to control cells, where myosin light chains are strongly phosphorylated at the trailing edge but excluded from the leading edge. This effect of Hem-1 depletion could reflect or cause the observed increased frequency of leading edge retraction (Figure 7C and Videos 7C and 7C2).

## Discussion

We have presented evidence that Hem-1–scaffolded protein complexes organize the leading edge and are required for neutrophil polarity and chemotaxis. These complexes include, but are not limited to, the well-known WAVE2 complex, which mediates Rac activation of actin polymerization. We find (Figures 2 and 3) that a substantial fraction of Hem-1 can be biochemically separated from WAVE, more than 60% of Hem-1–containing complexes do not associate with WAVE, and Hem-1 forms complexes with polarity regulating proteins other than WAVE. RNAi-induced disruption of Hem-1 produces multiple cytoskeletal, signaling, and polarity defects during chemotaxis.

Here we discuss possible roles of Hem-1–scaffolded complexes in controlling key events at the leading edge of polarized neutrophils, including (a) actin polymerization, (b) pseudopod-stabilizing positive feedback loops dependent and independent of F-actin, and (c) inhibition of myosin-based contraction at a location where it would otherwise inhibit F-actin–mediated protrusion. Some of these functions of leading edge complexes can be explained by known activities of the WAVE complex, while others cannot.

### Actin Polymerization and Protrusion of the Leading Edge

Actin polymerization at the neutrophil's leading edge substantially depends on Hem-1–containing complexes (Figure 4). This is very likely due to Hem-1′s role in regulating WAVE2, although we cannot exclude the possibility that Hem-1 might also regulate the actin cytoskeleton through direct binding to filamentous actin [46]. The actin polymerization defect of Hem-1–depleted cells is especially marked at low fMLP concentrations and at late times after stimulation. Only one other genetic manipulation in neutrophils, ablation of Rac activity, impairs actin polymerization so profoundly [11,64].

We note, however, that fMLP-dependent actin polymerization and polarity are not completely ablated in Hem-1–depleted cells (Figure 4). While residual functions at the leading edge of these cells may reflect activity of a small amount of residual Hem-1, it is also possible that fMLP regulates actin polymerization by mechanisms that do not include either Hem-1 or WAVE-family proteins and that may or may not involve Rac. In accord with the latter idea, genetic ablation of SCAR, the only WAVE homolog expressed of Dictyostelium discoideum, produces amoebae that retain considerable ability to polarize and migrate toward a source of chemoattractant [19,22]. Furthermore, genetic ablation of Nap1 (the Hem-1 homolog) in *Dictyostelium* produces stronger defects in cell adhesion and migration than ablation of SCAR (Robert Insall, personal communication), strongly supporting an evolutionarily conserved role of Hem1/Nap1 complexes in regulating cell processes above and beyond their role in WAVE/SCAR control. Thus it seems likely, as we propose below, that Hem-1–containing complexes play essential roles not simply in supporting attractant-stimulated actin polymerization but also in positive feedback loops that maintain stability of the pseudopod.

### Positive Feedback Loops at the Leading Edge

To explain the decreased fMLP-stimulated Rac activation and Akt/PKB phosphorylation in Hem-1–depleted cells (Figure 5), we propose that Hem-1 complexes play multiple roles in the Rac/ PIP_3_/actin positive feedback loop that amplifies chemoattractant signals needed to support robust actin polymerization at the leading edge [6,8–11]. Such feedback, which is essential for the persistence and polarized distributions of PIP_3_ and Rac activity, would be especially critical for responses to low concentrations of chemoattractant and at late time periods after its application, when receptor signals may have adapted to near basal levels. Failure to maintain this feedback should induce cells to revert to an unpolarized inactive state. Because depolymerization of actin decreases PIP_3_ generation in neutrophil-like HL-60 cells [9] and PIP_3_ is necessary for Rac activation [55,68,69], we are tempted to attribute part of the negative effect of Hem-1 depletion on fMLP-stimulated PIP_3_ generation and Rac activation to decreased formation of actin polymers. This cannot be the whole story, however, because Hem-1 depletion substantially diminishes both PIP_3_ generation and Rac activation even in latrunculin-treated cells, which are unable to polymerize actin (Figure 5D through 5F).

Which proteins in Hem-1–scaffolded complexes could account for this F-actin–independent feedback? One such protein is Abi-1, a Rac coactivator, which is found in Hem-1/Hem-2–scaffolded WAVE2 complexes [34,70]. Because Abi-1 forms a multiprotein complex that converts a Ras exchange factor into a Rac exchange factor [71,72], Rac activation of WAVE2 complexes might induce local generation of Abi-1/Rac exchange factor complexes, leading to a further increase in Rac activity. In this way, Rac would feed back positively to activate more Rac. To our knowledge, we present the first evidence suggesting that the WAVE2 complex or other leading edge complexes act both downstream and upstream of Rac. The Rac-mediated positive feedback loop appears to be separable from and acts in addition to the F-actin–based Rac/ PIP_3_/actin feedback loop. Both feedback loops are important for organizing the leading edge and both are dependent on Hem-1 complexes.

What is the role of leading edge complexes in PIP_3_ generation? PIP_3_ is known to act upstream of both Rac activation and actin polymerization, but Rac is also required for fMLP-stimulated PIP_3_ accumulation in neutrophils [10,11]—that is, Rac acts upstream of PIP_3_ as well. Thus Rac-to-Rac positive feedback might be necessary for maintaining PIP_3_ production. Alternatively, PIP_3_ production could be modulated by other components of leading edge complexes, such as Vps34, which is known to mediate PIP_3_ production in Schizosaccharomyces *pombe* [73].

### Exclusion of Rho-Myosin Signaling from the Front

Sharply differing morphologies and actin assemblies at the neutrophil's leading and trailing edges are thought to be maintained in part by localization of distinct signaling pathways—Rac and Rho-myosin, respectively—each of which locally inhibits the other [6,8–11,13,15]. A hint at mechanisms underlying one of these inhibitions comes from the observation (Figure 3) that Hem-1–scaffolded leading edge complexes associate with two sets of proteins that are known to inhibit Rho-myosin signals; these proteins include Rho GAPs, which inactivate Rho, and the regulatory and catalytic subunits of myosin light chain phosphatase, which inhibit myosin-mediated contractility. The possibility that Rac activates these inhibitory components of leading edge complexes merits further experiment. Such a mechanism could cooperate with the known ability of Rac to inhibit myosin activity by PAK-mediated phosphorylation and inactivation of myosin light chain kinase [74].

We also note that association with Hem-1–containing leading edge complexes could provide a mechanistic explanation for the genetic interaction, reported in C. elegans [75], between Rac and the regulatory subunit of myosin light chain phosphatase.

### Versatile Hem-1 Complexes Regulate Polarity at the Leading Edge

Our data strongly suggest that the functions of Hem-1 complexes are confined to roles at the leading edge of polarized neutrophils. Hem-1 complexes are located exclusively at the leading edge, and RNAi-mediated depletion of Hem-1 produces multiple defects at the leading edge in fMLP-treated cells: a decrease in polarized actin polymerization, aberrant morphology and persistence of pseudopods, decreased activation of Rac and PIP_3_, and a failure to exclude phosphorylated myosin (Figures 4, 5, 7C, and 7D). Superoxide production, an unpolarized response to fMLP that also depends on PIP_3_ generation and Rac activation, is not impaired in Hem-1–depleted cells (Figure 6). Remarkably, knockout of P-Rex1 (a leukocyte-enriched Rac activator) produces the opposite phenotype (highly defective superoxide but relatively normal chemotaxis [76,77]). These data suggest that different pools of Rac may regulate different effectors in neutrophils and that Hem-1 complexes are essential for the pool of Rac involved in chemotaxis and actin polymerization.

Why do WAVE2 and other potential Rac effectors associate with the Hem-1 scaffold, instead of binding directly to activated Rac? Certainly the scaffolded complexes can serve to position the effectors appropriately at the leading edge (although the mechanism for localizing the complexes themselves is unknown). A second possibility is that Rac and Nck, whose affinities for the WAVE regulatory complex led to discovery of the scaffold [35–37], are not necessarily its only (or even principal) inputs. A third possibility is that localized Hem-1–dependent scaffolds provide platforms for feedback loops, perhaps including but not limited to Abi-1–mediated Rac-to-Rac positive feedback [72], as noted above. Other separately scaffolded complexes that associate with the Hem1/CYFIP core such as fragile X mental retardation protein (FMRP) might locally regulate other processes such as protein translation [78,79].

**Figure 8:**
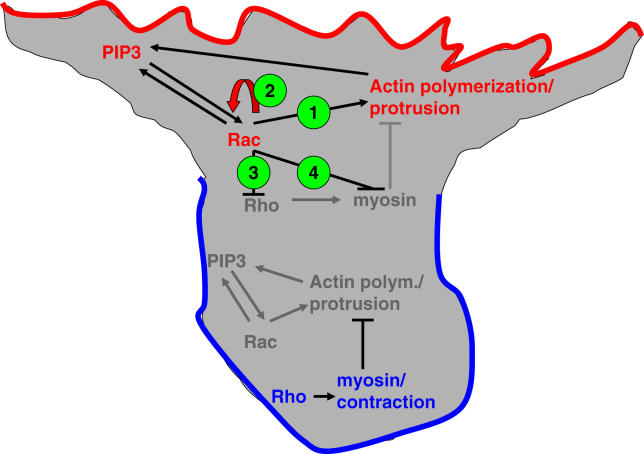
Role of Leading Edge Complexes in Cell Polarity Migrating cells make different actin assemblies in the front and back. The leading and trailing edges of the cell are organized by different GTPases. Rac plays a role in two positive feedback circuits that organize the leading edge and drive stable protrusion (we call this nested feedback circuit Rac/actin/PIP_3_ positive feedback). A separate GTPase (Rho) organizes myosin-based contraction at the trailing edge, primarily through inactivation of myosin light chain phosphatase, leading to local phosphorylation and activation of myosin light chain. Several positive and negative feedback circuits are essential for the proper organization of cell polarity, but not all of the proteins involved in these circuits are known. In this paper, we discuss several leading edge protein complexes that play a role in organizing of the leading edge. These leading edge complexes link Rac with actin assembly (Activity 1) and play a role in a Rac positive feedback circuit (Activity 2), both essential elements of the positive feedback loop that organizes the leading edge. Leading edge complexes also potentially link Rac to the inhibition of Rho activity (Activity 3) and inhibition of myosin activation (Activity 4), both of which would act to exclude myosin phosphorylation from the leading edge. For simplicity, this model focuses on activities that are addressed in this paper and omits other possible links involved in Rac/actin/PIP_3_ positive feedback and from Rac to Rho and myosin.

In summary, we propose that Hem-1 and CYFIP1/2 together scaffold protein complexes that convert information from multiple inputs into multiple integrated outputs at the leading edge. We imagine that Rac is only one of these inputs and that WAVE-stimulated actin polymerization is only one of several potential outputs; the latter could include local inactivation of the Rho/myosin–mediated contraction and facilitation of the Rac/PIP_3_/actin positive feedback that stabilizes the leading edge (Figure 8). Other inputs or effectors in leading edge complexes could impinge on regulation of Rho GTPases other than Rac and Rho (e.g., Cdc42), chemoattractant receptors, and heterotrimeric G proteins. Together, these effectors may set in motion an entire program of response that is coordinated at the leading edge during chemotaxis. Further experiments will focus on understanding how multiple upstream inputs regulate effectors in Hem-1–scaffolded complexes and on characterizing the composition of specific complexes responsible for the polarity and signaling functions that are disrupted in Hem-1–depleted cells.

## Materials and Methods

### Materials

Human fibronectin was from BD Biosciences Pharmingen (San Diego, California, United States). HSA (low endotoxin), BSA (low endotoxin), DMSO, and fMLP were from Sigma-Aldrich (St. Louis, Missouri, United States). Oligonucleotides were obtained from Integrated DNA Technologies (IDT) (Coralville, Iowa, United States). WAVE1, WAVE2, and WAVE3 antibodies were from Theresia Stradal's laboratory (Braunschweig, Germany). The anti–pan-WAVE rabbit polyclonal and Abi-1 mouse monoclonal antibodies were from Giorgio Scita's laboratory (Milan, Italy). The WAVE2 polyclonal goat antibody (C-14) was from Santa Cruz Biotechnology (Santa Cruz, California, United States). The pEN-hH1 and pL-UGIP plasmids were from Iain Fraser (Pasadena, California, United States). The pCMV8.91 and pMD.G plasmids were from William Seaman's laboratory (San Francisco, California, United States). The anti-Akt1 polyclonal antibody was from New England Biolabs (Beverly, Massachusetts, United States). All of our phospho-specific antibodies were from Cell Signaling Technology (Beverly, Massachusetts, United States): phospho-Akt/PKB (Thr308), phospho-myosin light chain 2 (Ser19), and phospho-PAK1 (Ser199/204)/PAK2 (Ser192/197) antibody, which is referred to as pSer199PAK throughout the text. The anti-tubulin mouse monoclonal was from Sigma-Aldrich. The Rac1 antibody was from BD Biosciences Pharmingen. Mouse and rabbit secondary antibodies conjugated to horse radish peroxidase were from Amersham Biosciences (Little Chalfont, United Kingdom). Rhodamine- and Alexa 647–conjugated phalloidin were from Molecular Probes (Eugene, Oregon, United States).

The Hem-1 peptide antibodies were generated in conjunction with Zymed (San Francisco, California, United States). Rabbits were injected with a mixture of an internal and a C-terminal antibody to human Hem-1 646–659 (CKQRQTPRKGEPERD) and 1114–1127 (CRNAYREVSRAFHLN). Each peptide antibody was independently affinity purified from the crude antisera. The human RhoGAP4 antibodies were generated as above but with 490–503 (CKSRQPRPSSQYNQR) and 933–946 (CASHPQGLDTTPKPH) of human RhoGAP4 used to generate the antibodies.

Cells were grown in full medium (RPMI-1640, 8% fetal calf serum, 1% penicillin-streptomycin) at a density of 0.2 to 1.8 × 10^6^ cells/ml. For differentiation, cells growing in log phase were diluted to a density of 0.1 × 10^6^ cells/ml in full medium containing 1.3% DMSO and grown for 6 to 7 days without changing the medium. Transient transfection was performed essentially as described in Srinivasan et al. [10].

### Vector construction

We tested the ability of several hairpins to knock down Hem-1 transiently expressed in 293 cells. Two constructs worked well in this assay and were used to generate lentiviruses that also contained CFP or GFP as a coinfection marker. One of these hairpins very effectively knocked down Hem-1 expression in differentiated HL-60 cells. To construct the hairpin RNAi cassette, two complementary DNA oligonucleotides were chemically synthesized, containing a 19-nucleotide sequence targeting the Hem-1 gene : 5′- GATCCCCGGACATACTTGTTCAGATCTTCAAGAGA GATCTGAACAAGTATGTCCTTTTTG-3′ and 5′- TCGACAAAAAGGACATACTTGTTCAGATCT CTCTTGAAGATCTGAACAAGTATGTCCGGG-3′.

After annealing, the hairpin was inserted into the pEN_hH1c plasmid between the BamHI and XhoI sites immediately downstream of the human H1 promoter. The lentiviral vector pDSL-hpUCIP was derived from the pL-UGIP vector described previously [81] by replacing the GFP with CFP (AgeI, BsrGI) to generate pL_UCIP and then inserting a Reading Frame A (RfA) cassette at the HpaI site. The LR clonase reaction (Invitrogen, Carlsbad, California, United States) was performed to insert the hH1 promoter-siRNAi hairpin construct at the RfA cassette into pDSL_hpUGIP and pDSL_hpUCIP. As controls, empty pL-UGIP and pL-UCIP plasmids were used for virus production.

### Lentivirus generation

HEK-293T cells at 90% confluence in a 75-cm^2^ flask were cotransfected by lipofection (Lipofectamine 2000; Invitrogen) with 20 μg of expression vector, 15 μg of packaging vector pCMV8.91, and 10 μg of VSV-G expression plasmid pMD.G (latter two from William Seaman's laboratory). The virus-containing supernatant was collected after 48 h and concentrated approximately 20-fold (Centriprep YM-50; Amicon, Millipore, Billerica, Massachusetts, United States). Then 100 to 200 μl of concentrated virus was added to 600,000 HL-60 cells in 1.5 ml of medium. Cells were amplified for several generations, and infected cells were sorted by FACS.

### Stimulated cell lysates/Western blots

For assaying protein phosphorylation in stimulated cells, we used a modification of the TCA protocol [82]. Differentiated HL-60 cells were starved for 1 h in modified Hanks' buffered saline solution (mHBSS: 150 mM sodium chloride, 4 mM potassium chloride, 1 mM magnesium chloride, 10 mM glucose, 20 mM HEPES [pH 7.4]—buffer A) containing 0.2% HSA, washed into calcium-free Gey's buffer containing 1 mM diisopropylfluorophosphate at a 1 to 2 million cells/ml, equilibrated for 10 min at 37 °C, and stimulated with 1/10 volume of 10× chemoattractant, and the reaction was stopped and proteins were precipitated by adding an equal volume of ice-cold 20% TCA containing 40 mM NaF and 20 mM β-glycerophosphate. Following TCA disruption, cells were incubated on ice for 20 min, spun at 14K in a 4 °C microfuge for 15 min, washed with chilled 0.5% TCA containing NaF and β-glycerophosphate, and resuspended in SDS sample buffer and boiled for 5 min.

For assaying Rac-GTP levels in HL-60 lysate, we performed PAK pulldown assays essentially as described [55] except that the HL-60 cells were prepared as above (prior to TCA addition) and PAK GBD was used on beads (20 μg of PAK GBD GST per assay point) instead of in suspension.

For Western blots, samples were separated on 4% to 12% gradient gels, transferred to pure nitrocellulose, blocked in Odyssey blocking buffer, incubated with 1:1,000 primary antibodies overnight at 4 °C in Odyssey blocking buffer plus 0.1% Tween-20, washed in TBST, incubated for 1 h in 1:2,000 to 1:4,000 Alexa 680 secondary antibodies, washed in TBST, then TBS, and imaged on an Odyssey infrared imaging system.

### Cell fixation, staining, and imaging

For fluorescence microscopy, cells were washed with RPMI and resuspended in mHBSS containing 1.8% HSA. The suspension was placed onto fibronectin-coated coverslips without washing, and cells were stimulated by addition of fMLP in mHBSS/HSA. The medium was quickly replaced with 3.7% paraformaldehyde in CSK/sucrose (10 mM HEPES [pH 7.2], 138 mM KCl, 3 mM MgCl_2_, 2 mM EGTA, 320 mM sucrose). After 10 min, the cells were solubilized with 0.2% Triton X-100 in CSK for 5 min and stained with rhodamine- or Alexa 647–conjugated phalloidin. The slides were mounted in Vectashield (Vector Labs, Burlingame, California, United States). Images were taken on a spinning disk confocal microscope (see below).

Fluorescent live cell imaging and some Nomarski time series were acquired on a Nikon TE2000U inverted microscope equipped with a Perkin Elmer (Wellesley, California, United States) Ultraview spinning disk confocal, a Solent Scientific (Segensworth, United Kingdom) 37 °C humidified incubation chamber, and a Hamamatsu (Hamamatsu City, Japan) Orca ER Cooled CCD camera.

For F-actin analysis by FACS, cells were washed and starved for 1 h in starving medium (RPMI, 0.25% BSA) and resuspended in mHBSS containing 0.5% HSA. Cells were stimulated by addition of fMLP, fixed by addition of paraformaldehyde (in PBS) to a final concentration of 3.7%, and placed on ice for 20 min. After centrifugation (5,000 rpm, 2 min), the cell pellet was resuspended in PBS, 5% milk, 0.1% Triton X-100, and Alexa 647–conjugated phalloidin (1:100); stained for 20 min; and washed twice in PBS, 0.1% Triton X-100. The sample was analyzed on a FACSCalibur machine. For visual analysis of cells stimulated in suspension, 1% HSA was used, cells were fixed by adding an equal volume of 7.4% paraformaldehyde in CSK, and the cells were stained with rhodamine-conjugated phalloidin (which exhibits a higher signal-to-noise ratio than other fluorescent phalloidins because rhodamine-phalloidin increases its fluorescence upon binding F-actin).

### Reactive oxygen assay

HL-60 cells that had been induced to differentiate into neutrophil-like cells by culture with DMSO for 6 days were resuspended in Dulbecco's PBS (with divalent cations and glucose plus 1 mg/ml low endotoxin BSA; Sigma A9306), and 100 μl was pipetted into 700 μl of assay buffer (Dulbecco's PBS plus 1 mg/ml BSA, 5.7 U/ml horseradish peroxidase [Type XII, Sigma P8415], and 150 μM luminol) prewarmed to 37 °C. After 5 min (time zero in the figure) in a luminometer at 37 °C (AutoLumat LB953; Perkin Elmer, Gaithersburg, Maryland, United States), assays were initiated by the introduction of agonist. For each experiment, equal numbers of control and knockdown cells were run in parallel for 100 nM, 10 nM, or 0 nM fMLP or 5 μg/ml PMA. The overall counts of fMLP-stimulated superoxide production were used as a multiplier between runs to normalize for variations in overall cell responsiveness.

### Preparation of high-speed cytosol

Pig leukocyte cytosol was prepared essentially as described [83]. Pig blood was obtained from Blood Farm (Groton, Massachusetts, United States). Then 18.2 l of blood was collected into a polypropylene jug containing 2.9 l of 1× sterile ACD anticoagulant (80 mM sodium citrate, 15 mM NaH2PO_4_*2H_2_O, 160 mM glucose, 17 mM citric acid, and 2 mM adenine). Blood was transported to the laboratory at room temperature. At the laboratory, 190 ml of 154 mM NaCl/3% polyvinylpyrrolidone (MW 360,000) was added per liter of blood plus anticoagulant, mixed thoroughly, poured into 2-l polypropylene containers, and allowed to settle into two phases for 30 to 45 min. The upper phase (containing leukocytes and contaminating red blood cells) was decanted and pelleted at 1,500*g* for 8 min at 15 °C in an IEC swingout rotor. The supernatant was poured off, and the pellets were resuspended in calcium-free mHBSS (buffer A) containing 0.1% HSA. Cells were pelleted tightly at 1,500*g* for 8 min and resuspended in buffer A.

Cells were resuspended in a minimum volume of buffer A, and then 20× volume of ddH_2_O was added for 20 s to lyse contaminating red blood cells. Then 1.1× volume 10× buffer A was added to regain an isotonic solution. Cells were pelleted and washed (repeating the osmotic shock step if necessary) and then resuspended in freshly prepared 3 mM diisopropylfluorophosphate to inactivate serine proteases and allowed to sit for 20 min on ice. Typically, 50 ml of packed leukocyte pellet or more is obtained per 20 l of blood. Cells were pelleted, and resuspended in relaxation buffer (100 mM KCl, 50 mM HEPES/KOH [pH 7.2] at 4 °C, 0.25 M sucrose, 2 mM EGTA, 1 mM MgCl_2_, 1 mM DTT, 0.1 mM PMSF, and 1× EDTA-free protease inhibitor tablets [Roche, Basel, Switzerland] per 25 ml of solution). Cells were cavitated in a nitrogen parr bomb (350 psi, 20 min) or minimally sonicated for 3 × 5 s at 50% constant output. Disrupted cells were spun at 900*g* for 10 min to remove nuclei and unbroken cells and then 160,000*g* or greater for 60 min to remove membranes. High-speed supernatant was carefully removed without disturbing the pellet. For purifications, the lysate was typically used fresh, but it was also stable when snap frozen in liquid nitrogen and stored at −80 °C for at least a year. This lysate is fully functional for PI(4,5)P_2_ and Cdc42-GTP-gamma-S–induced actin polymerization (data not shown). Lysates from 6-day differentiated HL-60 cells were prepared as above for pig leukocytes.

### Chromatography of lysates, WAVE2 complex purification

Leukocyte WAVE2 complex was purified by 0% to 40% ammonium sulfate precipitation and dialyzed into buffer S (100 mM NaCl, 1 mM MgCl_2_, 2 mM EGTA, 10% glycerol, 1% betaine, and 20 mM PIPES [pH 7.0]). The precipitate was resuspended and dialyzed into buffer S, loaded onto a high-performance HiTrap S (GE Healthcare, Amersham Biosciences) cation exchange column, and eluted with a linear 100 mM to 1 M gradient of NaCl on an AKTA FPLC. WAVE2 peak fractions were identified by Western blotting, pooled, dialyzed into buffer Q (buffer S with 20 mM Tris [pH 7.5] in place of PIPES), loaded onto a high-performance HiTrap Q anion exchange column, and eluted with a linear 100 mM to 1 M gradient of NaCl on an AKTA FPLC. WAVE2 peak fractions were pooled, dialyzed into buffer S, loaded onto a MonoS cation exchange column, and eluted with a linear gradient of 100 mM to 1 M NaCl on a Pharmacia (Uppsala, Sweden) SMART system. Peak fractions were pooled, loaded onto a Superose 6 column preequilibrated with buffer B (140 mM KCl, 10 mM NaCl, 1 mM MgCl_2_, 1% betaine, 10% glycerol, and 20 mM HEPES [pH 7.2]), and run in the same buffer. Thyroglobin, ferritin, catalase, and albumin were used as standards in a parallel run for the gel filtration column. Alternatively, Hem-1 antibodies were used to precipitate the WAVE2 complex prior to gel filtration.

### Immunoprecipitation

Affiprep protein A-Sepharose was incubated with affinity purified antisera or control rabbit IgG in TBST for 1 h at 4 °C, washed, and incubated with high-speed supernatant for 2 h at 4 °C with rotation. The beads were washed 2× with buffer B containing 300 mM KCl, 2× with buffer B containing 0.1% Tween-20, and 2× with buffer B, and then immunoprecipitated proteins were eluted with 0.2 mg/ml Hem-1 peptide in buffer B containing Roche EDTA-free protease inhibitors at 4 °C for 1 to 2 h. For some experiments, 100 mM glycine (pH 2.5) plus 100 mM NaCl was used to elute bound proteins for 10 min, and the samples were then neutralized with 1 M Tris (pH 9.5).

### Mass spectrometry analysis

Proteins identified in Figure 3A and in additional preparations were cut from the gels, reduced, and carboxymethylated (in gel) with 5 mM DTT and 55 mM iodoacetamide, washed with 50 mM ammonium bicarbonate (pH 8.5) and trypsin (Promega, Madison, Wiscosin, United States), digested, and extracted according to a previously published method [84]. Tryptic peptides were resuspended in buffer C (5% acetonitrile/0.2% formic acid) and pressure loaded offline onto a 100-μm (inner diameter) column packed with 12 cm of reverse phase Magic C18AQ (5 μm diameter, 200 Å pore size (Michrom Bioresources, Auburn, California, United States) material. The column was placed online with Agilent 1100 series HPLC binary pumps with an in-line flow splitter. Peptides were resolved with a gradient between 5% and 35% buffer D (95% acetonitrile/0.2% formic acid) at 60 bar over 30 min and subjected to analysis in XP ion trap mass spectrometer (Thermo Electron, San Jose, California, United States). Then 5 MS/MS spectra were acquired in a data-dependent manner from a preceding MS scan (400 to 1,800 *m/z*). All raw MS/MS spectra were searched nontryptically against the Human database from the National Center for Biotechnology Information (August 2004) by using the SEQUEST algorithm (AA). Nonstatic modifications were permitted to allow for the detection of oxidized Met (+16) and carboxymethylated CYS (+57). Only tryptic peptides were matched and filtered as described [84], which is as follows: (1) ΔCn score is at least 0.1; (2) for fully tryptic peptides, Xcorr score larger than 2.0, 1.8, and 3.4 for +1, +2, and +3 charge states, respectively. All proteins identified contained at least 3 fully tryptic peptides with the aforementioned parameters.

## Supporting Information

Figure S1 Only Trace Amounts of WAVE1 and WAVE3 Are Present in HL-60 Lysates Equal amounts of protein from brain lysate, control HL-60 lysate, and Hem-1 knockdown HL-60 lysate were blotted for antibodies to WAVE1, WAVE2, WAVE3, or pan-WAVE.
(99 KB PPT).

Figure S2 Gel Filtration of Control and Hem-1 Knockdown Lysates Equal amounts of protein from control or Hem-1 knockdown HL-60 cells were separated by gel filtration on a Superose 6 column and then blotted with antibodies to *son-of-sevenless*1/2, Hem-1, Rhogap4, Mypt1, or Rho guanine nucleotide dissociation inhibitor. The positions of molecular weight standards (thyroglobin, ferritin, catalase, and albumin) are noted. Note decreased high-molecular-weight pools of Rhogap4 in Hem-1 knockdown lysate.
(229 KB PPT).

Figure S3 Actin Depolymerization Abrogates the Disparity between Control and Hem-1 Knockdown Superoxide Production Equal numbers of control and Hem-1 knockdown cells were stimulated with 100 nM fMLP, and reactive oxygen production was monitored in a luminometer. Plots represent integrated intensity for 5 min following stimulation. Indicated cells were pretreated with 1 μM latrunculin B prior to stimulation.
(62 KB PPT).

Video S1 Hem-1 Strongly Polarizes following Uniform Stimulation of HL-60 Cells Differentiated HL-60 cells expressing GFP-tagged Hem-1 were exposed to uniform 20 nM fMLP (at t = 0 s) and imaged with a spinning disk confocal microscope at 5-s intervals at 37 °C. This video corresponds to Figure 1A.
(1.8 MB AVI).

Video S2 Control HL-60 Cells Persistently Polarize in Response to Uniform Stimulation Time-lapse Nomarski images of control HL-60 cells sandwiched between two coverslips and exposed to uniform 20 nM fMLP. This video corresponds to Figure 7B.
(1.6 MB WMV).

Video S3 Hem-1 Knockdown HL-60 Cells Exhibit Unstable Polarity in Response to Uniform Stimulation Time-lapse Nomarski images of Hem-1 knockdown cells sandwiched between two coverslips and exposed to uniform 20 nM fMLP. This video corresponds to Figure 7C.
(1.8 MB WMV).

Video S4 Hem-1 Knockdown HL-60 Cells Exhibit Unstable Polarity in Response to Uniform Stimulation Time-lapse Nomarski images of Hem-1 knockdown HL-60 cells sandwiched between two coverslips and exposed to uniform 20 nM fMLP. This is an additional example of the unstable leading and trailing edges of Hem-1 knockdown cells and does not correspond to a figure in the text.
(1.7 MB WMV).

### Accession Numbers

The GenBank (http://www.ncbi.nlm.nih.gov/Genbank) acession number for Hem-1 is BC001604.1. The AfCS-Nature Signaling Gateway (http://www.signaling-Gateway.org) codes for the plasmids discussed in this paper are pEN_hH1c plasmid (P05EENHH1CXG), pL-UGIP vector (L10GLUGIP1XA), and pDSL_hpUGIP (L11DDLUGIPXA).

## Abbreviations

Abi: Abelson interactor
CYFIP: cytoplasmic fragile X mental retardation interacting protein
dHL-60: differentiated HL-60
F-actin: filamentous actin
fMLP: formyl-methionyl-leucyl-phenylalanine
GAP: GTPase activating protein
GBD: GTPase binding domain
GFP: green fluorescent protein
Hem-1: hematopoietic protein 1
mHBSS: modified Hanks' buffered saline solution
Nap125: Nck-associated protein 125 kDa
PAK: p21-activated kinase
PIP_3_: phosphatidylinositol-(3,4,5)-*tris*-phosphate
PIR121: 121F-specific p53 inducible RNA
PKB: protein kinase B
RNAi: RNA interference
SCAR: suppressor of cyclic AMP receptor
TCA: trichloroacetic acid
WAVE: Wiskott-Aldrich syndrome protein family Verprolin-homologous protein
